# SLC16a6, mTORC1, and Autophagy Regulate Ketone Body Excretion in the Intestinal Cells

**DOI:** 10.3390/biology12121467

**Published:** 2023-11-26

**Authors:** Takashi Uebanso, Moeka Fukui, Chisato Naito, Takaaki Shimohata, Kazuaki Mawatari, Akira Takahashi

**Affiliations:** 1 Department of Preventive Environment and Nutrition, Institute of Biomedical Sciences, Tokushima University Graduate School, Tokushima 770-8503, Japan; 2 Faculty of Marine Biosciences, Fukui Prefectural University, Fukui 917-0003, Japan

**Keywords:** ketone body, solute carrier family 16 member 6: SLC16a6 (MCT7), mTORC1, autophagy, intestinal cell

## Abstract

**Simple Summary:**

Recently, it has become increasingly clear that ketone bodies have several functions, such as extending longevity, improving memory, increasing susceptibility to certain cancer therapies, and improving metabolic diseases such as obesity, hypertension, kidney disease, dyslipidemia, and non-alcoholic fatty liver disease. In addition, ketone bodies play a distinctive role in the intestinal epithelium, such as stem cell maintenance, cell proliferation and differentiation, and cancer growth. Through the present study, we found that SLC16a6, mTORC1, and autophagy involve ketone body excretion in the intestinal cells. A better understanding of the ketone body regulatory systems will facilitate the fine-tuning of normal and abnormal intestinal cell adaptation in homeostasis and injury.

**Abstract:**

Ketone bodies serve several functions in the intestinal epithelium, such as stem cell maintenance, cell proliferation and differentiation, and cancer growth. Nevertheless, there is limited understanding of the mechanisms governing the regulation of intestinal ketone body concentration. In this study, we elucidated the factors responsible for ketone body production and excretion using shRNA-mediated or pharmacological inhibition of specific genes or functions in the intestinal cells. We revealed that a fasting-mimicked culture medium, which excluded glucose, pyruvate, and glutamine, augmented ketone body production and excretion in the Caco2 and HT29 colorectal cells. This effect was attenuated by glucose or glutamine supplementation. On the other hand, the inhibition of the mammalian target of rapamycin complex1 (mTORC1) recovered a fraction of the excreted ketone bodies. In addition, the pharmacological or shbeclin1-mediated inhibition of autophagy suppressed ketone body excretion. The knockdown of basigin, a transmembrane protein responsible for targeting monocarboxylate transporters (MCTs), such as MCT1 and MCT4, suppressed lactic acid and pyruvic acid excretion but increased ketone body excretion. Finally, we found that MCT7 (SLC16a6) knockdown suppressed ketone body excretion. Our findings indicate that the mTORC1–autophagy axis and MCT7 are potential targets to regulate ketone body excretion from the intestinal epithelium.

## 1. Introduction

Ketone bodies, such as β-hydroxybutyrate (β-OHB), acetoacetic acid, and acetone, are produced during starvation, long-term exercise, or carbohydrate-restricted diet feeding and are used as an alternative energy source of glucose. When liver and muscle glycogens are depleted during starvation, fatty acids are transported from adipose tissue to the liver, where they are oxidized to produce acetyl CoA and ketone bodies [1]. The rate-limiting enzyme for ketone body synthesis is hydroxymethylglutaryl-CoA (HMG-CoA) synthase 2 (HMGCS2), which condenses acetoacetyl CoA and acetyl CoA to form HMG-CoA [2]. Ketone bodies produced in the liver are transported to the muscles, brain, and other tissues where they are converted to acetyl CoA and used as an energy source.

Recently, it has become clear that ketone bodies have roles other than an alternative energy source [3,4,5,6,7,8]. For example, it has been reported that β-OHB regulates the expression of genes involved in suppressing oxidative stress by inhibiting the class 1 histone deacetylase complex (HDAC) [4]. Other reports indicate that β-OHB regulates G protein-coupled receptor (GPR) 41 and GPR109A [5,6,7]. In addition, β-OHB inhibits the activation of NOD-like receptor protein 3 (NLRP3) inflammasome in macrophages [8]. Thus, β-OHB is assumed to have a specific role in local tissues and maintain systemic metabolic homeostasis as an energy source.

It has been reported that HMGCS2 mRNA and protein expressions are observed in the intestinal mucosal cells [9,10]. Recently, Wang Q et al. found that ketone bodies produced in the intestinal tract are involved in the differentiation of intestinal cells [11]. β-OHB stimulation or the overexpression of HMGCS2 induces Caco2 intestinal cell differentiation as noted by the increased expression of differentiation markers, such as cytokeratin 20 (KRT20) and p21Waf1. Conversely, the siRNA of HMGCS2 suppresses the differentiation of those cells. The knockdown of mammalian targets of rapamycin complex (mTORC) or the inhibition of mTORC by rapamycin upregulates protein expression of HMGCS2. In addition, HMGCS2 enriches intestinal stem cells and produces β-OHB. Moreover, HMGCS2 knockout to deplete β-OHB has been reported to reduce stemness, alter differentiation, and hamper regeneration of intestinal cells in mice [12]. These findings suggest that ketone bodies produced in intestinal epithelial cells may regulate nutrient metabolism and differentiation of intestinal epithelial cells or surrounding cells. However, the regulatory mechanism of ketone body concentration in intestinal epithelial cells remains unclear.

The intracellular and extracellular transport of monocarboxylic acids, such as β-OHB, is regulated by monocarboxylic acid transporters (MCTs). So far, fourteen MCTs and two sodium-dependent MCTs (SMCTs) have been identified [13,14,15,16]. Among them, MCT1, MCT2, MCT4, MCT7, and SMCT1 have been reported to transport β-OHB [13,15,17,18,19]. For example, it was reported that the Km values of MCT1, MCT2, MCT4, and SMCT1 expressed in Xenopus laevis oocytes for D-β-hydroxybutyrate are 10.1 mM, 1.2 mM, 130 mM, and 1.4 mM [13,17]. However, relative β-OHB levels in flow-sorted intestinal cells were around 20 to 125 nM/250,000 cells [12]; these concentrations might be pretty low compared with the Km value of the MCTs. Moreover, each MCT should competitively transport varieties of monocarboxylate that exist in different concentrations in cells [13,17]. Finally, the interaction between the production and transport of ketone bodies in intestinal cells is also unclear.

In the present study, we investigated the regulatory mechanism of ketone body concentration in intestinal epithelial cells. A better understanding of ketone body regulatory systems will facilitate the fine-tuning of normal and abnormal intestinal cell adaptation in homeostasis and injury.

## 2. Materials and Methods

### 2.1. Animals

Male C57BL/6J mice (8 weeks old) were purchased from a local breeding colony (Charles River Japan, Yokohama, Japan). The mice were housed in cages maintained at a constant temperature (23 ± 2 °C) with a 12 h light–dark cycle (8:00–20:00) and acclimatized for one week before experiment use. For the fasted sample collection, the mice fasted for 16 h (17:00 to 9:00). After exsanguination under anesthesia, mice tissue samples were harvested. The University of Tokushima Animal Use Committee approved the study (T28-84 and T2019-75), and the mice were maintained according to the National Institutes of Health guidelines for the care and use of laboratory animals.

### 2.2. Reagents

DMEM (GIBCO-A14430-01, phenol-red free, glucose free, pyruvate free, glutamine free) (DMEM(−/−)) was used as a fasting-mimicked reference medium for the stimulation of Caco2 and HT29 cells. Glucose (Nacalai tesque, Kyoto, Japan, 16806-25), sodium pyruvate (Tokyo Kasei Industry, Tokyo, Japan, P-5082), L-glutamine (Sigma-Aldrich, St. Louis, MO, USA, G7513), sodium octanoate (Sigma-Aldrich C5038), wy-14643 (Sigma-Aldrich C7081), torin1 (R and D Systems, Minneapolis, MN, USA, 4247/10), bafilomycin A (Cayman Chemical, Ann Arbor, MI, USA, 11038), chloroquine (Sigma-Aldrich 6628), torin2 (Selleck, Kanagawa, Japan, S2817), JR-AB2-011 (Med Chem Express, Monmouth Junction, NJ, USA, HY-122022), and 5-Aza-2’-deoxycytidine (Fujifilm Wako Pure Chemical Industries, Tokyo, Japan, 014-20943) were added to the medium at the indicated concentrations, and cells were stimulated for the indicated times.

### 2.3. Cell Culture

Caco2, HEK293, and HT29 cells were maintained and cultured in DMEM (Sigma-Aldrich D6429) containing 10% (*v*/*v*) fetal bovine serum (FBS, Thermo Fisher Scientific, Waltham, MA, USA, Gibco BRL) and 50 μg/mL gentamycin (Fujifilm Wako Pure Chemical Industries, 078-06061). The cells were passaged every 3–4 days in a CO_2_ incubator (5% CO_2_, 37 °C). For the experiments, cells were seeded in 60 mm dishes with 4 mL of a culture medium and 0.4 × 10^6^ cells, 35 mm dishes with 2 mL of a culture medium and 0.2 × 10^6^ cells, and 12 well dishes with 1 mL of a culture medium and 0.1 × 10^6^ cells. The ingredients of the reconstructed medium are shown in Supplementary Materials, Table S1.

### 2.4. Preparation of shRNA Constitutively Expressing Cell Lines

The oligonucleotide containing shRNA target sequences was determined by searching the Applied Biosystems website (Supplementary Materials, Table S2). The target sequence was cloned into a modified pEnter/U6 plasmid vector (Invitrogen, California, USA) containing the neomycin resistance gene from the pcDNA 3.1 plasmid vector (Invitrogen). After 1 day of passaging cells in 35 mm dishes, Caco2, HT29, or HEK293 cells were transfected with 2 μg of plasmid DNA and 2 μL of Lipofectamine 2000 (Invitrogen) in Opti-MEM (Thermo Fisher Scientific, GIBCO 31985062). After 4 h, 10% (*v*/*v*) of FBS was added. The medium was replaced with DMEM after 24 h, and the effect of the shRNA on the mRNA expression of each target gene was validated at 48 h post-transfection (Supplementary Materials, Figure S1). The effect of shRNA was confirmed in more than two cell lines, including HEK293, HT29, and Caco2. After 24 h of transfection, Caco2 or HT29 cells were then transferred into a 10 cm dish at 2 days post-transfection, and plasmid integrated cells were selected by adding 1 mg/mL of G418 (Fujifilm 078-05961). The cells were cultured for 21 to 28 days, and single-cell-derived colonies were stored. shLacZ-transfected cells were used as a control and confirmed mRNA or protein expression of the targeted gene.

### 2.5. mRNA Expression Analysis

Total RNA was extracted using standard methods as previously reported [20,21]. Total RNA from each sample was reverse transcribed into cDNA, and real-time quantitative PCR was performed to measure mRNA expression levels with a specific primer set (Supplementary Materials, Table S2). The mRNA expression levels were normalized with respect to expression levels of 18 s or β-actin.

### 2.6. Protein Extraction

Cultured cells were washed with ice-cold phosphate-buffered saline (PBS) and collected in a 1.5 mL tube. Cells were centrifuged at 5000× *g* for 1 min, and then the cell pellets were resuspended in a RIPA buffer (50 mM Tris-HCl, pH 7.4, 150 mM NaCl, 1% sodium deoxycholate, 0.1% sodium dodecyl sulfate (SDS)) with a protease inhibitor cocktail (PIC) (Nacalai 04080). Tissues were homogenized in a 10-fold volume of a RIPA buffer with a PIC. The resuspended cells or tissue homogenate were disrupted using a sonicator, and the samples were centrifuged at 12,000 rpm for 15 min at 4 °C. The supernatant was used for total cell fraction. To obtain cytosolic and membrane fractions, we used the Subcellular Protein Fractionation Kit (Thermo Fisher 78840) according to the manufacturer’s protocol. Samples were stored at −30 or −80 °C until use.

### 2.7. Protein Concentration Measurement and Western Blotting

The protein concentration of each sample was determined according to the method of the BCA protein assay kit (Thermo Fisher 23225). Protein samples were heated at 95 °C for 5 min in a sample buffer (200 mM Tris-HCl (pH 6.8), 2% SDS, 5% 2-mercaptoethanol, 10% glycerol) and subjected to SDS/PAGE. The proteins were separated via electrophoresis and transferred onto PVDF membranes (Immobilon-P, Merk Millipore, Massachusetts, USA). The membranes were blocked with a 1% skim milk TBS-T solution (20 mM Tris-HCl, pH 7.4, 150 mM NaCl, 0.05% Tween20) for 30 min and washed with TBS-T for 10 min 3 times and incubated with primary antibody (rabbit anti-HMGCS2 antibody (Abcam, Cambridge, UK, 137043), rabbit anti-phospho-p70 S6 kinase (T389) antibody (Cell signaling, Massachusetts, USA 9234), rabbit anti-EGF receptor antibody (Cell signaling 2232), rabbit anti-p70 S6 kinase antibody (Cell signaling 2708), rabbit anti-HSP90 antibody (Cell signaling 4877), rabbit anti-MCT1 antibody (Proteintech, Rosemont, IL, USA, 22787-1-AP), rabbit anti-MCT4 antibody (Proteintech 20139-1-AP), rabbit anti-basigin antibody (Proteintech 11989-1-AP), and mouse anti-β-actin antibody (Santa Cruz Biotechnology, Dallas, TX, USA, H1914) overnight at 4 °C. After the reaction was completed, the membranes were washed with TBS-T for 10 min × 3 times, and secondary antibodies (anti-IgG mouse-HRP MBL (458) and anti-IgG rabbit-HRP MBL (330)) were incubated for 1 h at room temperature. The membranes were then washed with TBS-T for 10 min 3 times and incubated with an enhanced chemiluminescence solution (GE Healthcare, Chicago, IL, USA), and chemiluminescence was detected using a LAS-3000UVmini (Fujifilm Wako Pure Chemical Industries). The band image was analyzed using Image J (version 1.53a).

### 2.8. Measurement of Ketone Body Concentration

After each experiment, the culture supernatant of cells was collected, and the extracellular ketone body concentration was measured. Cellular proteins were extracted with 100 μL of a RIPA buffer, and protein concentration was measured. Cell protein extracts were heated at 95 °C for 1 min and centrifuged at 12,000 rpm for 15 min. Intracellular ketone body concentration was measured in the supernatants of protein extracts. Ketone body concentration was determined using the Total Ketone body assay kit (Fujifilm) according to the manufacturer’s instructions. Extracellular and intracellular ketone body concentration was adjusted via protein concentration.

### 2.9. Capillary Electrophoresis (CE)–Mass Spectrometry (MS) and pH Analysis

Metabolite concentrations were measured via capillary electrophoresis (CE)–mass spectrometry as previously described [20,21]. The pH of the medium was determined using a pH meter (Horiba, Kyoto, Japan, D-51).

### 2.10. Statistical Analysis

All results are presented as mean ± standard error. A *t*-test or a Mann–Whitney U test was used for comparisons between two groups, the analysis of variance method was used for comparisons between four groups, and Tukey’s multiple comparisons were used to test for two-factor interaction. The significance level was set at *p* < 0.05. Excel-Tokei 2010 or SPSS (Statistics 25) were used for statistical analysis.

## 3. Results

### 3.1. HMGCS2 Expression and Ketone Body Concentration in Mouse Colons

**Figure 1:**
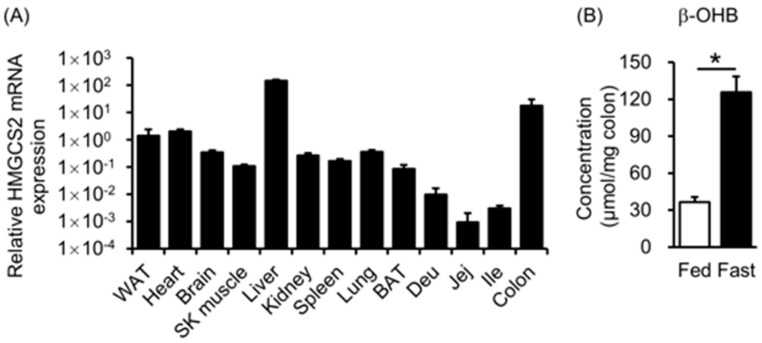
HMGCS2 expression and ketone body concentration in mouse colons. (**A**) Relative HMGCS2 mRNA expression levels adjusted by β-actin mRNA expression in indicated tissues. WAT: white adipose tissue, SK muscle: skeletal muscle, BAT: brown adipose tissue, Deu: duodenum, Jej: jejunum, Ile: ileum. (**B**) β-hydroxybutyrate (β-OHB) concentration in the colon at fed and fasted states. A *t*-test was used for comparisons between the two groups. (**B**) *n* = 3, *: *p* < 0.05.

To confirm physiological changes in ketone body metabolism, we first compared the expression of the rate-limiting enzyme of ketogenesis in the colon. HMGCS2 expression was higher in the colon than in other tissues, except for the liver (Figure 1A). β-OHB concentration in the colon was increased by fasting for 16 h (Figure 1B). These results suggest that ketone body metabolism in the colon was actively regulated in physiological conditions.

### 3.2. Ketone Body Production in Colorectal Cells

**Figure 2:**
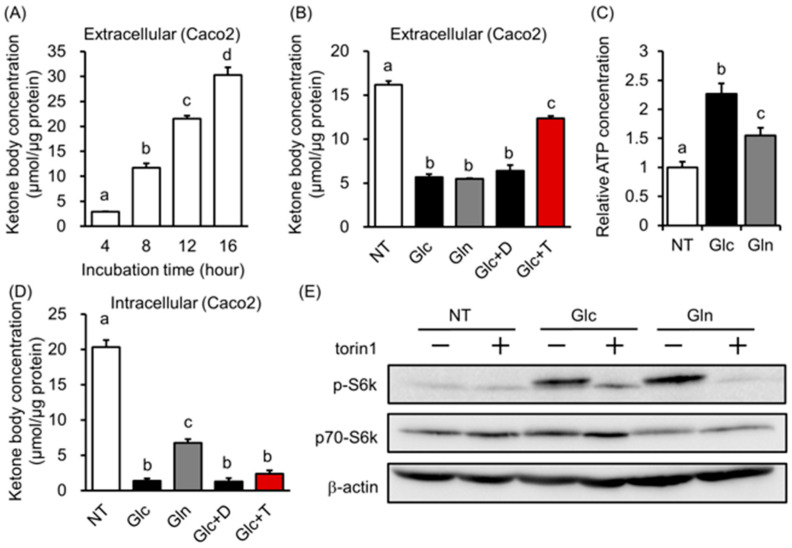
Ketone body production and excretion were regulated by energy status in Caco2 cells. (**A**) Changes in total ketone body concentration in the medium incubated with a fasting-mimicked medium for the indicated time. (**B**,**D**) Changes in total ketone body concentration in the medium (**B**) or cells (**D**) incubated with a fasting-mimicked medium (NT) with 20 mM of glucose (Glc), 2 mM of glutamine (Gln), 20 mM of glucose and 0.1% DMSO (Glc + D), or 20 mM of glucose and 1 μM of torin1 (Glc + T) for 12 h. (**C**) Changes in the cellular ATP level of a fasting-mimicked medium (NT), NT with 20 mM of glucose (Glc) or 2 mM of glutamine (Gln)-treated cells (NT as 1.0). (**E**) Band pattern of p70-S6 kinase, phosphorylated p-70 S6 kinase, and β-actin of cells treated with a fasting-mimicked medium (NT), NT with 20 mM of glucose (Glc) or 2 mM of glutamine (Gln) with or without 1 μM of torin1 for 12 h. The analysis of variance method was used for comparisons among groups, and Tukey’s multiple comparisons were used to determine which groups differed. (**A**–**D**) *n* = 3. The different letters indicate significant differences (*p* < 0.05).

To analyze the effect of starvation on ketone body metabolism in intestinal epithelial cells, Caco2 and HT29 cells were cultured in a fasting-mimicked medium without glucose, pyruvate, and glutamine. When Caco2 cells were incubated in this medium, extracellular ketone body concentration increased over time (Figure 2A). This increase was suppressed by the addition of glucose (Glc), pyruvate (Pyr), or glutamine (Gln) without changes in HMGCS2 expression (Figure 2B, Supplementary Materials, Figure S2A–C). In contrast, previously reported ketogenic media containing octanoate or peroxisome proliferator-activated receptor α (PPARα) agonist wy-14643 alone and a combination of those did not affect extracellular ketone body concentration and HMGCS2 expression (Supplementary Materials, Figure S2D,E) [22]. To clarify the differences in the intracellular metabolic state caused by a fasting-mimicked medium or glucose, pyruvate, and glutamine stimuli, 102 metabolites were comprehensively measured via CE-MS analysis. Principal component analysis showed that fasting, glucose, pyruvate, and glutamine stimulation had different metabolic states (Supplementary Materials, Figure S2F). In particular, glucose and glutamine stimulation showed similar trends in extracellular ketone body concentrations, whereas the metabolic states did not. Among the metabolites measured, an increased ATP concentration and a decreased AMP/ATP ratio, an energy status indicator was observed in the addition of glucose or glutamine compared with fasting-mimicked medium (Figure 2C and Supplementary Materials, Figure S2G). We also found that cellular ATP levels were negatively correlated with intracellular ketone body concentration (Figure 2D). From these results, we focused on the mammalian target of the rapamycin complex (mTORC), which reflects cellular energy status, and examined the relationship between mTORC and ketone body metabolism. Protein expression of the phosphorylation of S6 kinase (p-S6K), which reflects mTORC activation, was increased by glucose and glutamine stimulation, similar to the extracellular ketone body concentration (Figure 2E). The link between ketone body production and mTORC activation was confirmed in HT29 cells (Supplementary Materials, Figure S2H,I). We also confirmed that non-metabolizable glucose analog 2-deoxyglucose (2-DG) did not suppress ketone body excretion (Supplementary Materials, Figure S2J). To examine mTORC and ketone body metabolism in detail, we tested intracellular and extracellular ketone body concentrations following mTORC inhibition. Extracellular but not intracellular ketone body concentration was increased in intestinal cells treated with glucose and the mTORC inhibitor torin1 compared with glucose alone, suggesting that mTORC suppressed ketone body excretion (Figure 2B,D and Supplementary Materials, Figure S2H). Because torin1 inhibits both mTORC1 and mTORC2, to investigate the involvement of mTORC1 and mTORC2 in ketone body metabolism, Caco2 cells were stimulated with glucose, the mTORC1 inhibitor torin2, and the mTORC2 inhibitor JR-AB2-011. The extracellular ketone body concentration was increased in the torin2 treatment similar to the torin1 treatment, whereas it did not increase in the JR-AB2-011 treatment (Supplementary Materials, Figure S2K). These results indicate that ketone body production linked with cellular energy status and the export of ketone bodies are regulated at least in part in an mTORC1-dependent manner.

### 3.3. Autophagy Regulates Ketone Body Excretion in Colorectal Cells

**Figure 3:**
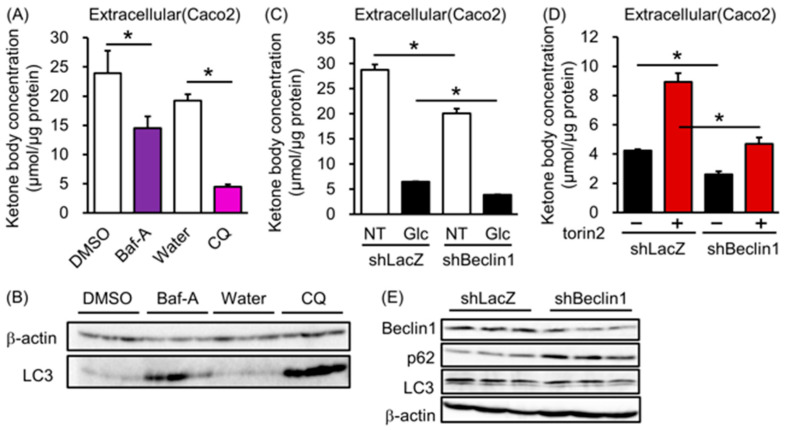
Effect of autophagy on ketone body excretion in Caco2 cells. (**A**) Changes in extracellular ketone body concentration (**A**) and LC3 and β-actin protein expression (**B**) of Caco2 cells incubated in a fasting-mimicked medium with 0.1% DMSO, 200 nM of bafilomycin A (Baf A), double distilled water (water), or 50 nM of chloroquine (CQ) for 16 h. (**C**,**D**) Changes in ketone body concentration of shLacZ or shBeclin1-transfected Caco2 cells incubated in a fasting-mimicked medium (NT) or 20 mM of glucose (Glc) and 20 mM of glucose with or without 1 μM of torin 2 for 16 h. (**E**) Changes in protein expression in shLacZ- or shBeclin1-transfected Caco2 cells. *T*-tests were used for comparisons between each vehicle control group (DMSO or water) and treated groups (BafA or CQ) (**A**). A *t*-test was used for comparisons between shLacZ and shBeclin1 cells treated in the same medium. (**C**,**D**) *n* = 3 *: *p* < 0.05.

It has been reported that the activation of mTORC1 inhibits autophagy [23,24]. Therefore, we next investigated whether autophagy is involved in ketone body metabolism in intestinal epithelial cells. Treatment cells with the autophagy inhibitors bafilomycin A (BafA) and chloroquine (CQ) suppressed the starvation-induced increase in extracellular ketone body concentration concomitant with accumulation of autophagy maker LC-3 (Figure 3A,B and Supplementary Materials, Figure S3A). We also found that the knockdown of beclin1, a key molecule in the autophagic machinery, partially reduced extracellular ketone body concentration in the starvation-mimicked medium with the accumulation of autophagy substrate p62 and a reduction in LC3 but did not reach statistical significance (Figure 3C,E and Supplementary Materials, Figure S3B–E). In this situation, intracellular ketone body concentration was not different between shLacZ cells and shBeclin1 cells (Supplementary Materials, Figure S3F). In addition, extracellular ketone body concentration was partially increased via the pharmacological inhibition of mTORC in beclin1 knockdown or chloroquine treatment cells (Figure 3D and Supplementary Materials, Figure S3G). These results suggest that the mTORC and autophagy pathways were additively associated with ketone body excretion in intestinal cells.

### 3.4. Basigin-Associated MCT Involves Ketone Body Import in Colorectal Cells

**Figure 4:**
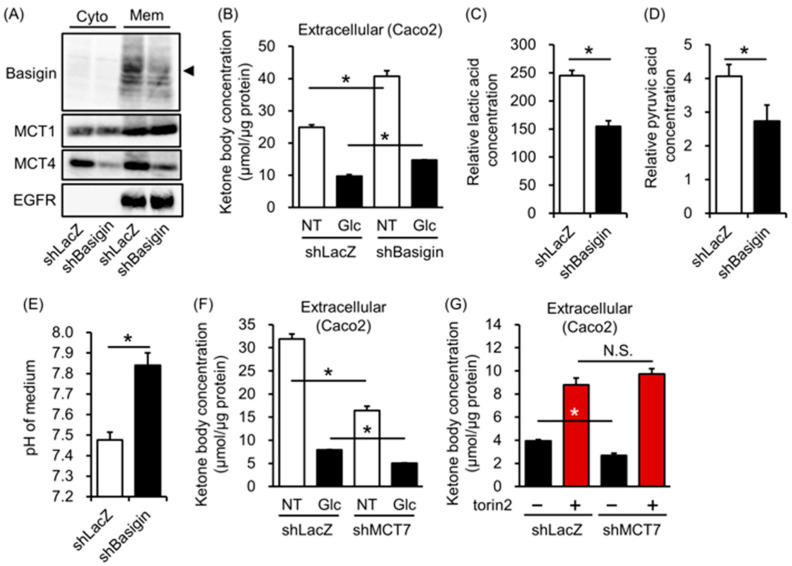
Effect of a monocarboxylate transporter on ketone body excretion in intestinal cells. (**A**–**E**): Changes in cytosolic (Cyto) and plasma membrane (Mem) protein expression (**A**); extracellular ketone body concentration (**B**); extracellular lactic acid concentration (**C**); pyruvic acid concentration (**D**); and pH of the medium (**E**) in shLacZ and shBasigin-transfected cells. (**F**,**G**) Changes in extracellular ketone body concentration in shLacZ- and shMCT7-transfected cells. Caco2 cells were incubated in a fasting-mimicked medium (NT), 20 mM of glucose (Glc), and 20 mM of glucose with or without 1 μM of torin 2 for 16 h. *T*-tests were used for comparisons between shLacZ and shBasigin or shMCT7 cells treated in the same medium. (**B**–**G**) *n* = 3 *: *p* < 0.05.

To further explore reliable ketone body transporters, we focused on MCTs. Among ketone transporters, we excluded SMCT1 (SLC5a8) and MCT2 (SLC16a2) because the gene expression of SLC5a8 and embigin, a binding partner of MCT2 [25], were suppressed by DNA methylation (Supplementary Materials, Figure S4A). To investigate sodium-dependent ketone body transport in colorectal cells, we reconstructed a starvation-modified medium in which sodium was replaced by N-methyl-D(-)-glucamine (NMDG). In this reconstructed medium, extracellular ketone body concentration did not change with the sodium-based starvation medium (Supplementary Materials, Figure S4B), suggesting that the sodium-dependent transporter did not involve ketone body transport. Next, we investigated the knockdown of basigin, the binding partner for MCT1 and MCT4, on ketone body transport [26]. Basigin and MCT4 expression were reduced in the membrane fraction of basigin knockdown cells but not MCT1 protein expression (Figure 4A). Surprisingly, extracellular but not intracellular ketone body concentration increased more in shBasigin cells than shLacZ cells, both in starved and unstarved conditions (Figure 4B and Supplementary Materials, Figure S4C,D). In addition to this, the knockdown of MCT1 did not affect extracellular ketone body concentration (Supplementary Materials, Figure S4E). In contrast, extracellular lactate and pyruvate concentrations were lower in shBasigin cells concomitant with a higher medium pH (Figure 4C–E and Supplementary Materials, Figure S4F). MCT4 knockdown cells also showed higher pH in the medium compared with the medium of shLacZ cells (Supplementary Materials, Figure S4G). These results suggest that basigin-associated MCT accelerates lactate and pyruvate and suppresses ketone body export in intestinal cells.

### 3.5. MCT7 Involves Ketone Body Export in Colorectal Cells

Finally, we found that extracellular ketone body concentration was reduced in the MCT7 knockdown of both Caco2 and HT29 cells without changing intracellular ketone body concentration in the fasting-mimicked medium (Figure 4F, Supplementary Materials, Figure S4H,I). Glucose supplementation additively reduced extracellular ketone body concentration (Figure 4F, Supplementary Materials, Figure S4I). However, when cells were treated with glucose and torin2, there was no difference in extracellular ketone body concentration between MCT7 knockdown cells and control cells (Figure 4G). These results suggest that the mTORC1 signal-mediated ketone body efflux partially associates with the MCT7-mediated ketone body efflux.

## 4. Discussion

**Figure 5:**
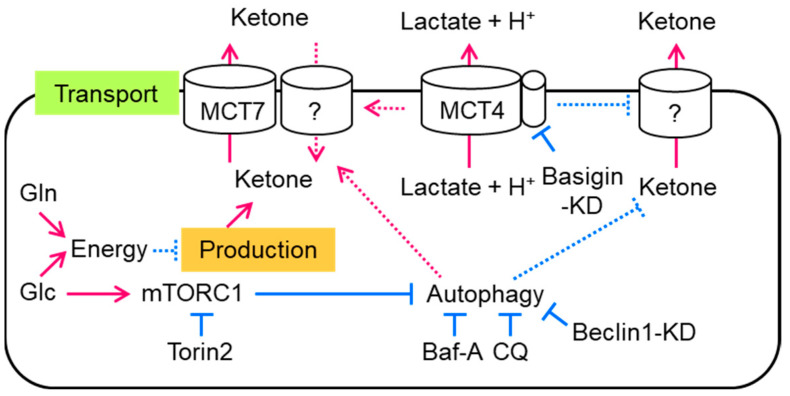
Summary of the regulatory mechanism of ketone body concentration in intestinal cells. The continuous arrows and lines show our study’s results and methods, and the discontinuous arrows and lines show unknown mechanisms that regulate ketone body transport in intestinal cells. Red arrows show acceleration, and blue lines show suppression. KD: knockdown.

In the present study, we found that a major fraction of ketone body production and excretion was independently regulated in intestinal cells (Figure 5). Ketone body production was suppressed by the supplementation of energy sources, such as glucose or glutamine. In addition, mTORC1 was activated by glucose-suppressed ketone body excretion. On the other hand, the inhibition of mTORC1 recovered a fraction of the excreted ketone bodies without changing ketone body production. Autophagy and MCT7 accelerated ketone body excretion. Unlike lactate or pyruvate, basigin-associated MCTs suppressed ketone body excretion.

In the fasting-mimicked medium, ketone body production was increased, and it was suppressed by the addition of glucose and glutamine but not 2-deoxy glucose, suggesting that an increase in energy source in the cell suppressed ketone body production. In fact, intracellular ketone body concentration was negatively associated with cellular ATP levels (Figure 2C,D). In this context, octanoate and a PPARα agonist, a transcriptional regulator of HMGCS2, did not suppress extracellular ketone body concentration. These results suggest that the contribution of the octanoate to the energy source is negligible in Caco2 cells. In addition, HMGCS2 protein expression was not different among NT, Glc, Pyr, and Gln groups, suggesting that HMGCS2 activity might be regulated by post-translational modifications in our experiment (Supplementary Materials, Figure S2B) [27,28,29]. Moreover, the inhibition of mTORC1 increased extracellular ketone body concentration but did not increase intracellular ketone body concentration. These results indicate that mTORC1 signals may suppress ketone body export. Further studies are needed to reveal the regulatory system of ketone body production.

Among ketone body transporters, such as MCT1, MCT2, MCT4, MCT7, and SMCT1, SMCT1 (SLC5a8) and MCT2 chaperon embigin expression were turned off in intestinal cells (Supplementary Materials, Figure S4A) [30,31]. In addition, basigin-associated MCTs accelerated lactic acid and pyruvic acid excretion and suppressed ketone body excretion. The transport of substrates by MCTs coupled with proton transport is driven by proton gradients [13]. Therefore, the reduction in proton and lactic acid transport observed in basigin and MCT4 knockdown cells could affect ketone body transport indirectly. In addition, we found that ketone body excretion was reduced in the MCT7 knockdown of both Caco2 and HT29 cells. This reduction was masked by mTORC1 inhibition. Two possibilities for the masking effect of the mTORC1 signaling-mediated ketone efflux in MCT7 knockdown cells are the unrevealed interaction between the mTORC1 pathway and MCT7 or the complementary role of other transport carriers. Further studies are needed to clarify the mechanism involving basigin-associated ketone body export and the regulatory mechanism between mTORC1 and MCT7.

A recent study reported that β-OHB infused into the blood could detect cecum content [12]. This result indicates that an increased concentration of β-OHB should be derived from not only colon tissue but also the liver through the blood. In addition, secreted β-OHB from colonic epithelial cells potentially compromises gut microbiota composition and function. Some bacteria produce Poly-(R)-3-hydroxybutyrate (PHB) as a biopolymer consisting of linear chains of β-OHB and PHB, which is considered to serve as a carbon and energy store in these organisms [32]. These reports suggest that β-OHB excreted from intestinal cells may have a role in gut microbiota.

There are some limitations in our study. For example, we mainly used Caco2 and HT29 cells as a model of epithelial cells. Both cells are derived from colorectal cancer cells and generally, cancer cells and normal cells have different properties, such as the expression of genes involving a ketone body, fatty acid metabolism, and MCTs. In fact, SMCT1 and embigin expression were suppressed in those cells. Nevertheless, we showed the mTORC1–autophagy pathways and MCT7 regulating ketone body excretion, and these results may help us understand the regulatory mechanism of ketone body metabolism to fine-tune normal and abnormal intestinal cell homeostasis.

## 5. Conclusions

In the present study, we showed that ketone body production was increased by the exclusion of glucose, pyruvate, and glutamine from the medium. It was suppressed by the addition of glucose, pyruvate, or glutamine concomitant with an increase in ATP levels. We also found that the mTORC1–autophagy axis and MCT7 are at least partly involved in ketone body excretion in intestinal cells. In contrast, basigin-associated transporters totally regulated lactate excretion and ketone body uptake. These results clearly indicate that cellular ketone body concentration is regulated by several factors in intestinal cells (Figure 5).

## Data Availability

The data generated or analyzed during this study are provided in this published article and supplemental information.

## Supplementary Materials

The following supporting information can be downloaded at https://www.mdpi.com/article/10.3390/biology12121467/s1, Figure S1: Effect of shRNA; Figure S2: Changes in ketone body concentration, metabolites, and HMGCS2 expression in Caco2 cells in different conditions; Figure S3: Effect of Beclin1 knockdown on autophagy-related protein and intracellular ketone body concentration; Figure S4: Effect of monocarboxylate transporter on ketone body excretion in colorectal cells; Figure S5: Full Western blots; Table S1: Ingredients of the reconstructed medium; Table S2: Primers.
