# Supplementary material for: SLC16a6, mTORC1, and Autophagy Regulate Ketone Body Excretion in the Intestinal Cells
Source: Biology (Basel). 2023 Nov 26;12(12):1467. doi: 10.3390/biology12121467 (PMC10740559; doi:10.3390/biology12121467)
Supplement: Supplementary file 1 [file biology-12-01467-s001.zip › Rev Supplemental Figures.pdf]

Supplemental Materials

Title: SLC16a6, mTORC1, and autophagy regulate ketone body excretion in the intestinal cells

Takashi Uebanso[1,*], Moeka Fukui[1], Chisato Naito[1], Takaaki Shimohata[1,2], Kazuaki Mawatari[1], Akira Takahashi[1]

1. Department of Preventive Environment and Nutrition, Institute of Biomedical Sciences, Tokushima University Graduate School, Tokushima, Japan.
2. Faculty of Marine Biosciences, Fukui Prefectural University, Fukui, Japan.
*Correspondence: uebanso@tokushima-u.ac.jp Tel.: +81-88-633-9598

E-mail address: uebanso@tokushima-u.ac.jp
Phone: +81-88-633-9598; fax: +81-88-633-7092.
Department of Preventive Environment and Nutrition, Institute of Biomedical Sciences, Tokushima University Graduate School, 3-18-15, Kuramoto, Tokushima 770-8503, Japan.

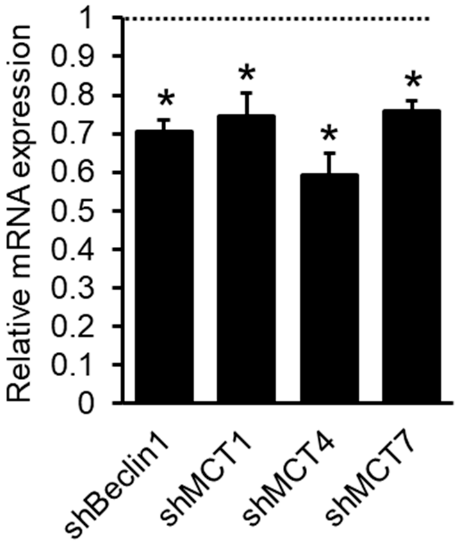

Supplemental Figure 1. Effect of shRNA

Changes in mRNA expression of each gene for specific shRNA. shRNA was transiently expressed in HEK293 cells and confirm mRNA expression of each target gene. T-tests were used for comparisons between shLacZ- and each shRNA-treated group. n =3 *: P < 0.05.

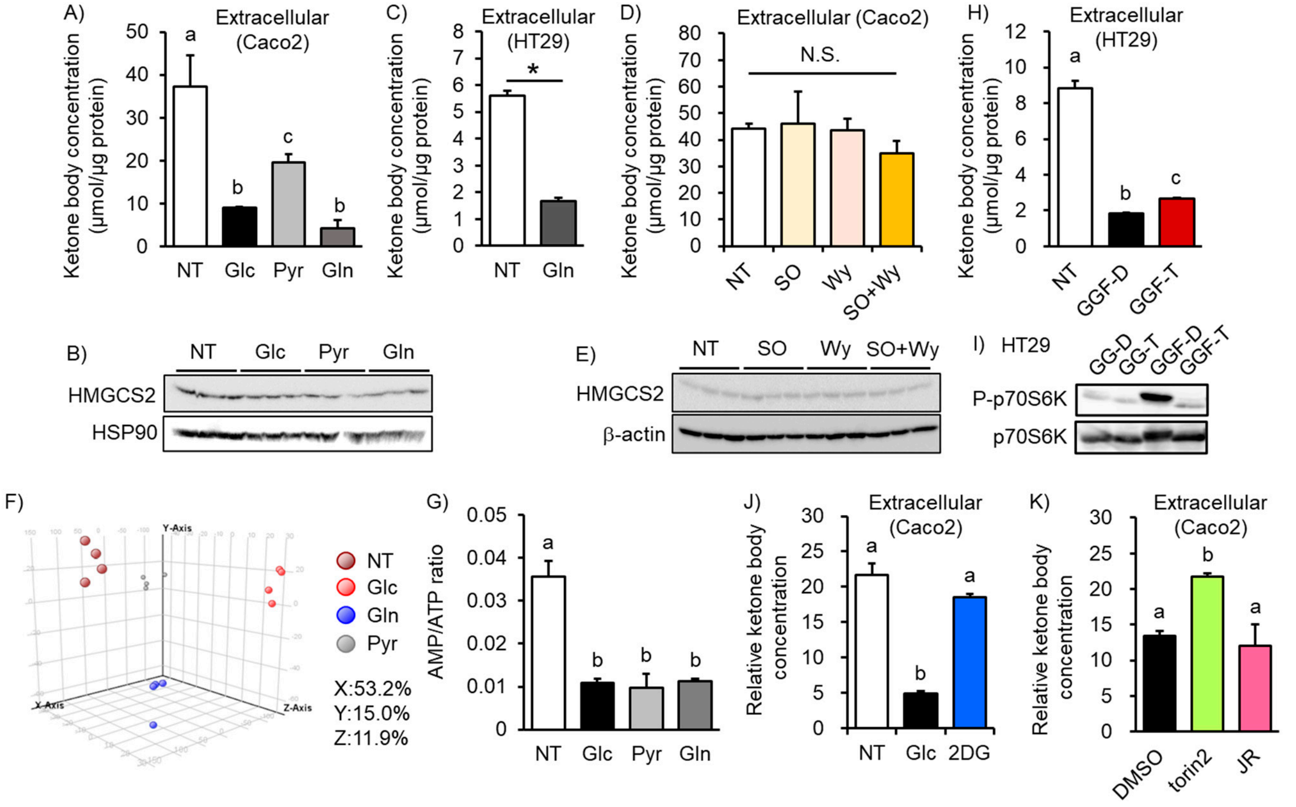

Supplemental Figure 2. Changes ketone body concentration, metabolites, and HMGCS2 expression in Caco2 and HT29 cells in different condition

(A, F, G) Changes in total ketone body concentration in medium (F), principal component analysis of

intracellular metabolites (G, and intracellular AMP/ATP ratio incubated with fasting-mimicked medium (NT), NT with 20 mM of glucose, NT with 10 mM of pyruvate, and NT with 2 mM of glutamine for 16 hours. (B, E) Changes in HMGCS2, β-actin, and HSP90 protein expression in Caco2 cells in indicated stimulation for 16 hours. NT: fasting-mimicked medium, Glu: NT with 20 mM of glucose, Pyr: NT with 10 mM of pyruvate, Gln: NT with 2 mM of glutamine liver, SO: 2 mM of sodium octanoate, Wy: 1 µM of wy-14643, and SO + Wy: 2 mM of sodium octanoate and 1 µM of wy-14643. (C, D, H, J, K) Changes in total ketone body concentration in medium incubated with indicated stimulation for 16 hours (D, K) or 12 hours (C, H, J). GGF-D: 20 mM of glucose, 2 mM of glutamine, 10% FBS, and 0.1 % DMSO, T: 1 µM of torin2. 2DG: 20 mM of 2-deoxy glucose, torin2: 20 mM of glucose with 1 µM of torin2, JR: 20 mM of glucose with 1 µM of JR-AB2-011. (I) Band pattern of p70-S6 kinase, phosphorylated p-70 S6 kinase, and total p-70 S6 kinase of HT29 cells treated with with 20 mM of glucose (G) and 2 mM of glutamine (G) with or without 10% FBS (F), DMSO (GG-D, GGF-D), or 1 µM of torin2 (GG-T, GGF-T) for 12 hours. The analysis of variance method was used for comparisons among groups, and Tukey's multiple comparisons were used to determine which groups differed. (A, D, G, H, J, and K). A-t-test was used for comparisons between the two groups (C). n =3. The different letters indicate significant differences ($p < 0.05$).

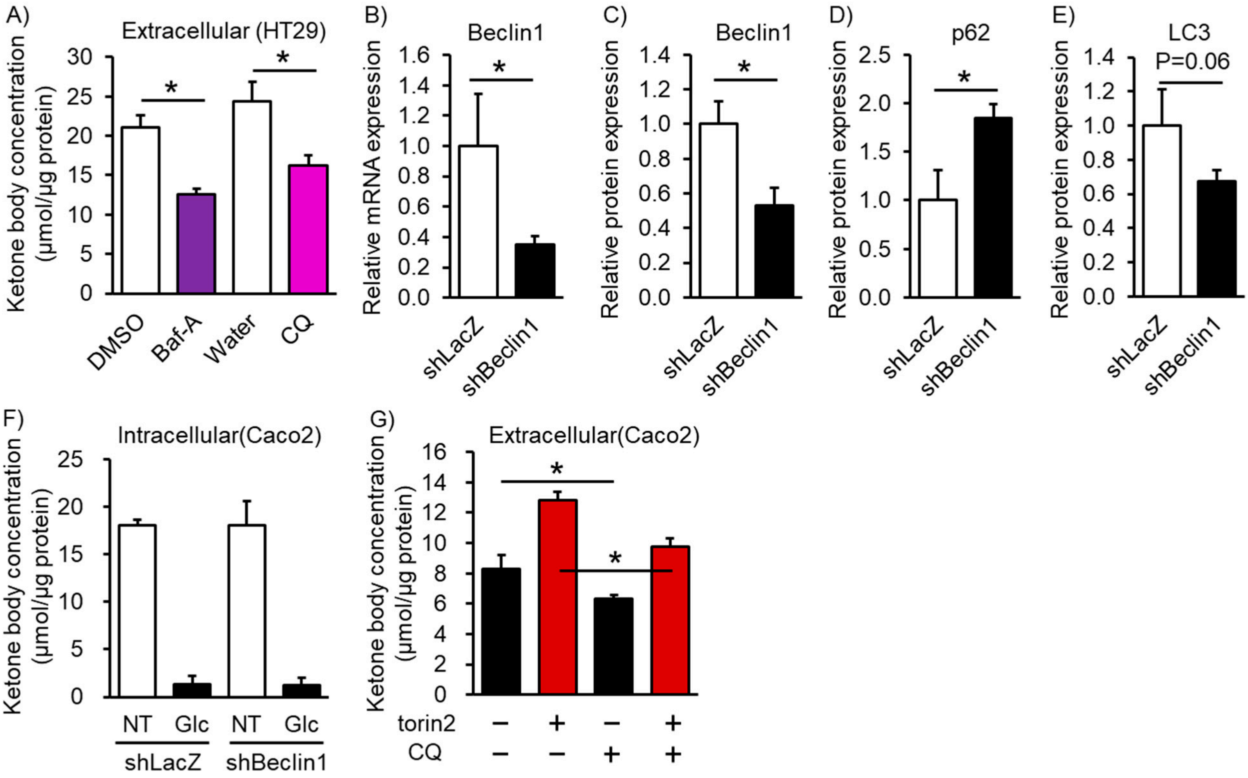

Supplemental Figure 3. Effect of Beclin1 knockdown on autophagy-related protein and ketone body concentration

(A) Changes in extracellular ketone body concentration of HT29 cells incubated in fasting-mimicked medium with 0.1 % DMSO, 200 nM of bafiromycin A (Baf A), double distilled water (Water), or 50 nM of chloroquine (CQ) for 16 hours. (B-E) Changes in Beclin1 mRNA (A), Beclin1 (B), p62 (C),

and LC3 (D) protein expression in shLacZ and shBeclin1 cells. (F, G) Changes in intracellular ketone body concentration in shLacZ and shBeclin1 cells stimulated with fasting-mimicked medium (NT) or with 20 mM glucose (Glc) (F) or 20 mM of glucose with or without 1 µM of torin2 and 50 nM of chloroquine (CQ). T-tests were used for comparisons between each vehicle control group (DMSO or Water) and treated groups (BafA or CQ) (A). T-tests were used for comparisons between shLacZ and shBeclin1 cells treated in the same medium (B-F). T-tests were used for comparisons between with or without torin2-treated cells and with or without CQ-treated cells (G). n =3 *: $P < 0.05$.

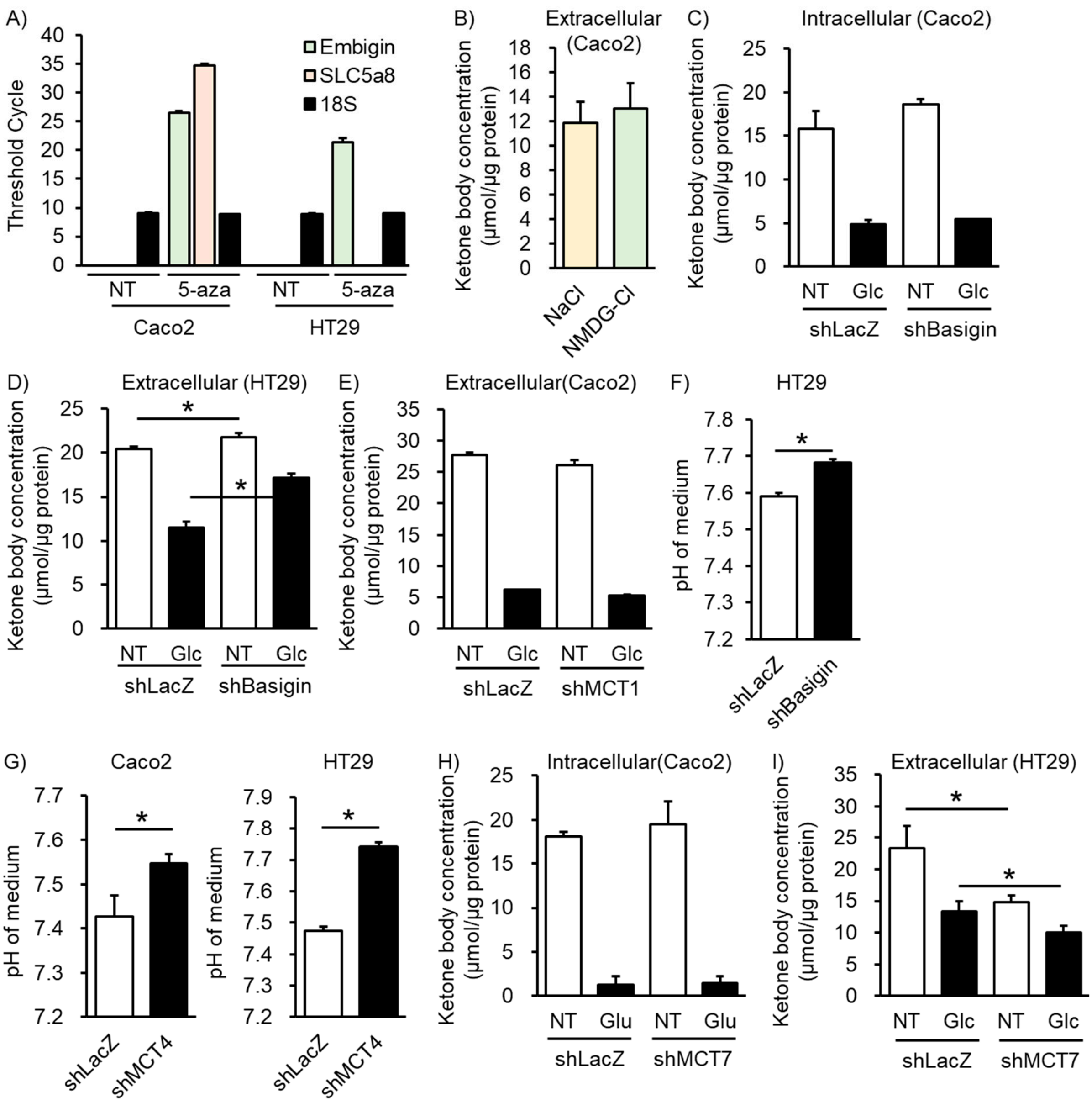

Supplemental Figure 4. Effect of monocarboxylate transporter on ketone body excretion in colorectal cells

(A) Changes in Embigin, SLC5a8, and 18S mRNA expression in Caco2 and HT29 cells treated with 5-aza deoxy cytidine for 4 days. (B) Changes in ketone body concentration in medium incubated

with reconstructed medium (Supplemental Table1) for 12 hours. NMDG: N-Methyl-D(-)-glucamine. (C-E) Changes in ketone body concentration in shLacZ and shBasigin Caco2 cells (C), HT29 cells (D) or shLacZ and shMCT1 Caco2 cells (E). (F, G) Changes in pH of cultured medium in shLacZ, shBasigin or shMCT4 cells. (H, I) Changes in ketone body concentration in shLacZ and shMCT7 cells. A t-test was used for comparisons between NaCl and NMDG-Cl treated cells (B). T-tests were used for comparisons between shLacZ and each shRNA-treated cell in the same medium (C to I). n =3 *: $P < 0.05$.

Figure. 2E

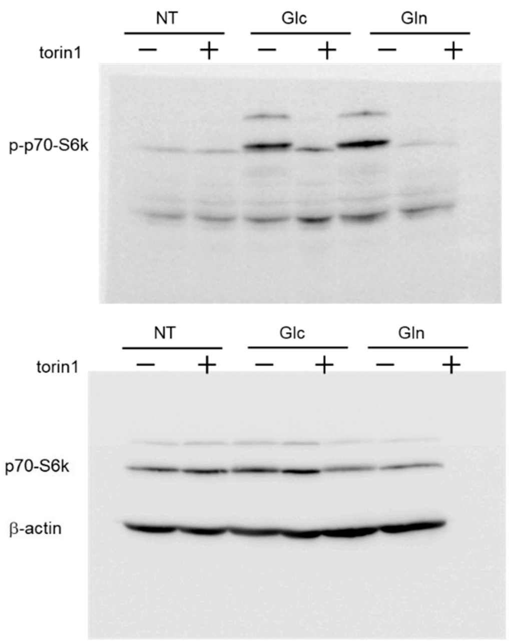

Figure. 3B

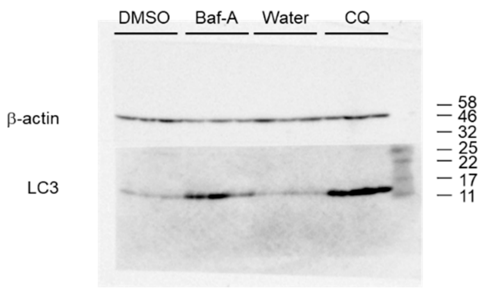

Figure. 3E

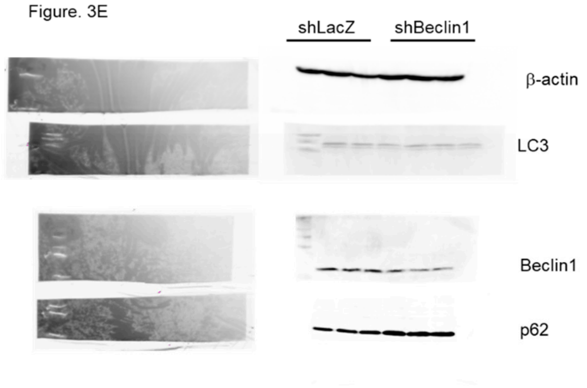

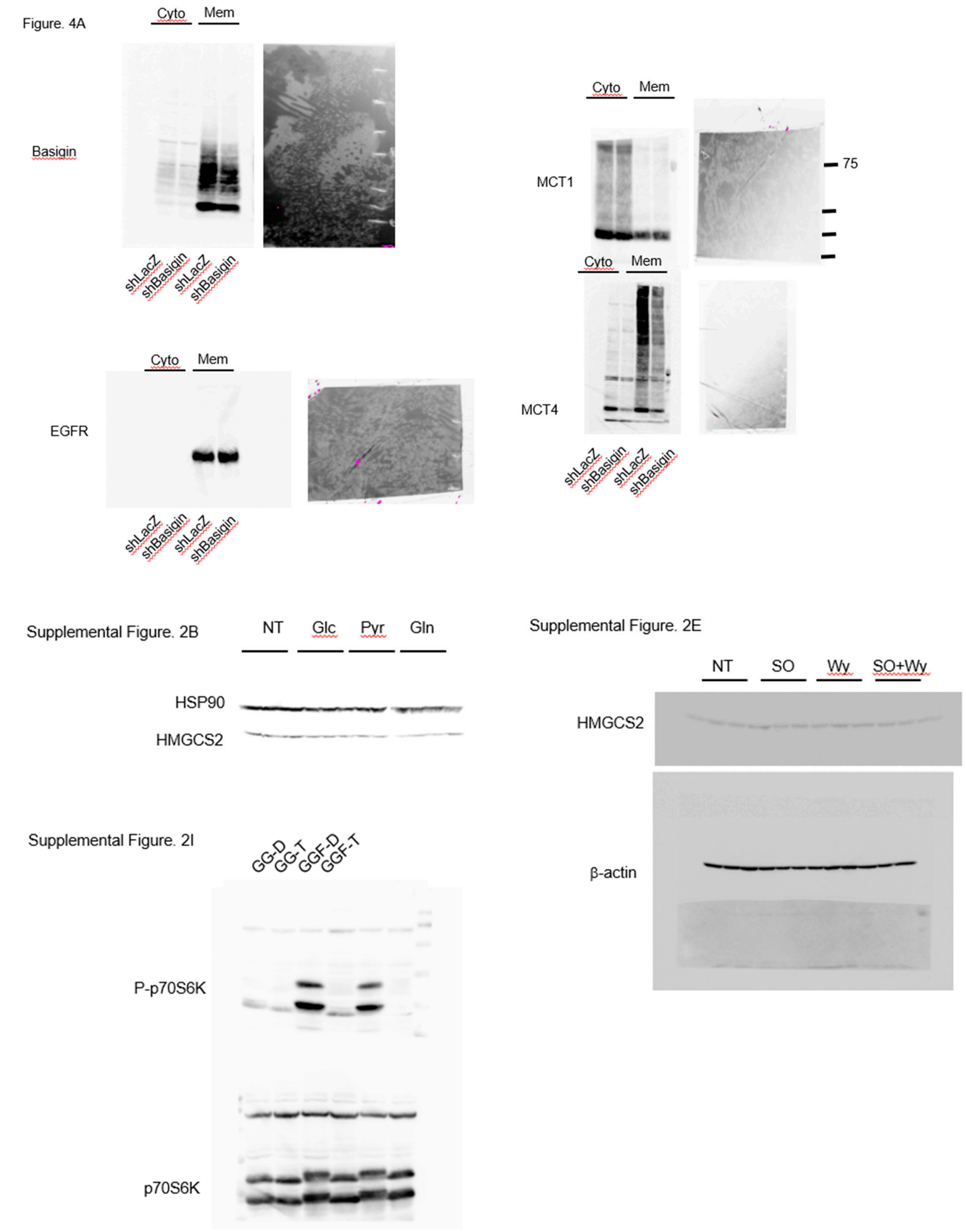

Supplemental Figure 5. Full western blot images
