# Supplementary material for: SLC16a6, mTORC1, and Autophagy Regulate Ketone Body Excretion in the Intestinal Cells
Source: Biology (Basel). 2023 Nov 26;12(12):1467. doi: 10.3390/biology12121467 (PMC10740559; doi:10.3390/biology12121467)
Supplement: Supplementary file 1 [file biology-12-01467-s001.zip › Supplemental Table 1.pdf]

Supplemental Table 1 Ingredients of the reconstructed medium

	DMEM	Reconstructed medium
Inorganic salts (mM)		
$CaCl_2$	1.8	-
$CaHCO_3$	-	1.8
$Fe(NO_3)_3$	0.0002	-
$FeCl_2$	-	0.0002
$MgSO_4$	0.8139	-
$MgCl_2$	-	0.8139
KCl	5.3333	5.3333
$NaHCO_3$	44.0	-
NaCl or NMDG-Cl	110.3 (NaCl)	140
NaH_2PO_4	0.9058	-
KH_2PO_4	-	0.44
K_2HPO_4	-	0.44

Reconstructed buffer contains MEM Vitamin Solution (Thermo Fisher), MEM Non-Essential Amino Acids Solution (Thermo Fisher), MEM Amino Acids Solution (Thermo Fisher), and 10 mM HEPS (2-[4-(2-hydroxyethyl)piperazin-1-yl]ethanesulfonic acid). pH of medium was adjusted 7.4 by KOH. NMDG: N-Methyl-D-glucamine.
