# Supplementary material for: SLC16a6, mTORC1, and Autophagy Regulate Ketone Body Excretion in the Intestinal Cells
Source: Biology (Basel). 2023 Nov 26;12(12):1467. doi: 10.3390/biology12121467 (PMC10740559; doi:10.3390/biology12121467)
Supplement: Supplementary file 1 [file biology-12-01467-s001.zip › Supplemental Table 2.pdf]

Supplemental Table 2 Primers

Primer	Sequence	Description
shSLC16a1-F	CACCGATGGATATCCTCCATAATGTTCGAAAACATTATGGAGGATATCCAT	NM_003051.4<br>NM_001166496.2
shSLC16a1-R	AAAAATGGATATCCTCCATAATGTTTTCGAACATTATGGAGGATATCCATC	
shSLC16a4-F	CACCGCTGGTTATATGATTATACCCACGAATGGGTATAATCATATAACCAG	NM_004696<br>tv1 to 6
shSLC16a4-R	AAAACTGGTTATATGATTATACCCATTCGTGGGTATAATCATATAACCAGC	
shSLC16a6-F	CACCGCACTGCTCAGACCCATCTTTACGAATAAAGATGGGTCTGAGCAGTG	NM_001174166.2<br>NM_004694.5
shSLC16a6-R	AAAACACTGCTCAGACCCATCTTTATTCGTAAAGATGGGTCTGAGCAGTGC	
shBasigin-F	CACCGCAGGATTCTGTTCCTTAGGTTCGAAAACCTAAGGAACAGAATCCTG	NM_001728.4<br>tv1 to 5
shBasigin-R	AAAACAGGATTCTGTTCCTTAGGTTTTCGAACCTAAGGAACAGAATCCTGC	
shBeclin1-F	CACCGCAGCAGTTCAAAGAAGAGGTTCGAAAACCTCTTCTTTGAACTGCTG	NM_003766.5<br>NM_001313998.2
shBeclin1-R	AAAACAGCAGTTCAAAGAAGAGGTTTTCGAACCTCTTCTTTGAACTGCTGC	
SLC5a8-F	AGCCCTGTGTATTGGAATGG	NM_145913.5
SLC5a8-R	CAGACCAACAAGTGCTCCAA	
Embigin-F	TGTGGTGCTGAGCTATTTGG	NM_198449.3
Embigin-R	TTTCCCCTCATCTGAGTGCT	
β-actin-F	TGTTACCAACTGGGACGACA	NM_007393.5
β-actin-R	GGGGTGTTGAAGGTCTCAAA	
Basigin-F	AGCACCAGAATGACAAAGGC	NM_001728.4
Basigin-R	AATCTACGGGGTGGGTTTTC	tv1 to 5
Beclin1-F	ACAGTGGACAGTTTGGCACA	NM_003766.5
Beclin1-R	TCCGTAAGGAACAAGTCGGT	tv1 to 4
SLC16a1-F	GTCATGATTGTTGGTGGCTG	NM_003051.4
SLC16a1-R	CAATCATGGTCAGAGCTGGA	NM_001166496.2
SLC16a4-F	GGGTTGCTGATCAAAACTGG	NM_004696
SLC16a4-R	GCAAAGAAACTGGCAAGTCC	tv1 to 6
SLC16a6-F	ACACTGGGATTCTTTGCACC	NM_001174166
SLC16a6-R	TCCCTGTTGAGGACAAAACC	NM_004694.5
hHMGCS2-F	TATAAGGGGCTGGAGGCTTT	NM_005518.4
hHMGCS2-R	GCATCCTGGGATACTCGAAA	NM_001166107.1
mHMGCS2-F	CCCCTGAGGAATTCACAGAA	NM_008256.4
mHMGCS2-R	GCATCTCATCCACTCGTTCA	

18S-F	AAACGGCTACCACATCCAAG	NR_003278.3
18S-R	GGCCTCGAAAGAGTCCTGTA	NR_145820.1

Underlines indicate target sequence and complement sequence. tv: transcript variant, HMGCS2: 3-hydroxy-3-methylglutaryl-CoA synthase 2
